# Occurrence of *Staphylococcus aureus, Staphylococcus epidermidis,* and *Staphylococcus pseudintermedius* colonization among veterinarians in the province of Malaga, Spain

**DOI:** 10.14202/vetworld.2024.2719-2724

**Published:** 2024-12-06

**Authors:** Fernando Fariñas-Guerrero, Antonio J. Villatoro, Eduardo Martinez-Manzanares, Rosa López-Gigosos

**Affiliations:** 1 Institute of Clinical Immunology and Infectious Diseases, 29010 Málaga, Spain; 2 Cátedra One Health. Málaga University and Official College of Veterinarians, 29010 Málaga, Spain; 3 Immune Stem (Immunology and Cell Therapy), 29018, Málaga, Spain; 4 Department of Microbiology, Faculty of Medicine, Málaga University, 29010, Málaga, Spain; 5 Department of Public Health and Psychiatry, Faculty of Medicine, Málaga University, 29010, Málaga, Spain

**Keywords:** antibiotic resistance, drug-resistant *Staphylococcus aureus*, one health, seroprevalence, *Staphylococcus* colonization, veterinarians

## Abstract

**Background and Aim:**

*Staphylococcus pseudintermedius* and *Staphylococcus aureus* are common colonizing pathogens in companion animals. These opportunistic pathogens can cause infections of varying frequency and severity in humans and pets. Studies on *Staphylococcus* colonization in veterinarians are scarce. This study aimed to investigate the colonization of the nostrils and hands by *S. aureus, Staphylococcus epidermidis*, and *S. pseudintermedius* among healthy clinical practice veterinarians in the province of Malaga (Spain), with a particular focus on their potential antibiotic resistance.

**Materials and Methods:**

A request for voluntary participation was extended to professionals from the Official College of Veterinarians of Malaga. Nasal and hand swabs were collected by two trained technicians in January 2024, and all samples were delivered to the laboratory within 24 h. Gram staining, catalase, oxidase, and coagulase tests were performed. The susceptibility of the isolated bacteria to 11 antibiotics was evaluated.

**Results:**

A total of 50 clinical practice veterinarians were enrolled in the study, comprising 36 women and 14 men from 31 veterinary clinics across Málaga province. A total of 32% of the nasal samples yielded *S. aureus*, whereas 64% were found to contain *S. epidermidis*. In total, 30% of the hand samples yielded *S. aureus* and 30% yielded *S. epidermidis*. The participants did not exhibit any strains of *S. pseudintermedius* in their nasal samples or hands. Two strains (11.1%) of methicillin-resistant *S. aureus* were isolated from 18 strains isolated from nostrils. Furthermore, a high prevalence of *S. aureus* strains resistant to ampicillin (94.4%) and amoxicillin (72.2%) was observed.

**Conclusions:**

The colonization profiles of veterinary professionals were similar to those observed in the general population. Further research is required among veterinary professionals, companion animals, and their owners to better understand the colonization processes and the pet-human interface within a “One Health” approach.

## Introduction

The One Health initiative is a global strategy for fostering collaboration across all aspects of health care, including human, animal, plant, and environmental health. Over the past few decades, the roles of pets and companion animals have evolved, with an increasing emphasis on social function. Pets can play an important role in their owners’ physical and mental health, but they can also pose a risk of zoonotic infection. It is imperative to assess the positive and negative aspects of the human-pet relationship, with a particular focus on zoonotic aspects, particularly in industrialized countries [1]. From this perspective, antimicrobial resistance (AMR) is a critical global threat to human and animal health, and pets play an important role [2]. Nasal and hand colonization among healthcare professionals is critical for controlling hospital infections [3]. *Staphylococcus pseudintermedius* and *Staphylococcus aureus* are common colonizing pathogens in companion animals. It is, therefore, of significant interest to gain a more detailed understanding of staphylococcal colonization among veterinary professionals [4]. *S. aureus* is a commensal microorganism of the human skin and mucous membranes. However, it is also a frequent cause of serious infections, with high morbidity and mortality rates and associated healthcare costs [5–10]. Nasal colonization has been demonstrated to play a pivotal role in the pathogenesis of infections by this bacterial species in patients undergoing surgery [11–13], dialysis [11, 14], and intensive care unit admission [15]. Methicillin-resistant *S. aureus* (MRSA) is the most commonly isolated multidrug-resistant pathogen in many parts of the world. The rapidly increasing rates of healthcare-associated and community-acquired MRSA represent significant clinical, public health, and economic challenges [16, 17]. The transmission of MRSA occurs through direct contact with the infected individual, indirect contact with an infected individual’s environment (e.g., doorknobs and handrails), indirect contact with an infected individual’s fomites (e.g., towels, bedding), and indirect contact with an infected individual’s pets [18–20]. *S. pseudintermedius* is a species belonging to the *Staphylococcus intermedius* group, a group to which species such as *Staphylococcus intermedius* and *Staphylococcus delphini* [21] and is the most frequent causative agent of canine bacterial infections [22]. Methicillin-resistant *S. pseudintermedius*, which exhibits multiple resistance phenotypes, has emerged globally as a nosocomial pathogen frequently isolated in veterinary hospitals [23]. Although there are few documented cases, *S. pseudintermedius* has been isolated from human infections, primarily through dog bites [24, 25]. It can cause severe disease, including septicemia [26]. Currently, there is a paucity of studies on the prevalence of human nasal colonization with *S. pseudintermedius*. Han *et al*. [27] observed a prevalence of 3%–4.5% in humans and 25%–65.9% in dogs. Similarly, Rodrigues *et al*. [28] revealed that *S. pseudintermedius* was present in 1.5% of veterinary professionals, whereas MRSA was identified in 14.7% of the same group.

The potential risk of staphylococcal transmission from dogs to humans and vice versa was also demonstrated. Furthermore, the clinical relevance of *S. pseudintermedius* transmission from dog to human and *S. aureus* transmission from human to dog should not be underestimated [29]. *Staphylococcus epidermidis* is a member of the normal microbiota of human skin and mucous membranes, along with other coagulase-negative staphylococci. It is typically a commensal organism present in biological samples without clinical consequences. However, its ability to create biofilms (the main virulence factor) in devices such as catheters and mechanical heart valve prostheses makes this species one of the most common causes of nosocomial infections. It is also associated with other diseases, including endocarditis and sepsis. The latter is particularly prevalent in neonates and immunocompromised patients. Most strains are multidrug-resistant, largely due to the expression of the PBP2a protein, which confers resistance to methicillin, a mechanism shared with certain strains of *S. aureus* [30].

It is postulated that the professional group of veterinarians may exhibit a distinct colonization of *Staphylococcus* species, which is derivative of their typical occupational contact with pets and other animals. Furthermore, we intend to examine the AMR of these microorganisms and determine whether they diverge from those of the general population with reference to the most recent publications on the subject [31].

This study aimed to determine the level of colonization of the nasal mucosa and hand skin by *S. aureus, S. epidermidis*, and *S. pseudintermedius* in a population of healthy clinical practice veterinarians and volunteers working in different veterinary centers in the province of Malaga. In addition, this study investigated the AMR.

## Materials and Methods

### Ethical approval and Informed consent

This study was approved by the One Health Chair Commission of the University of Malaga and the College of Veterinarians in October 2023 (approval number OHVET0004-2023). In November 2023, the voluntary participation of members of the Malaga Veterinary Association was requested to obtain nasal samples and hand swabs for microbiological study. Before their inclusion in the study, all volunteers were required to sign an informed consent form. All participants were informed that the results and data analysis would be anonymous.

### Study period and location

The study was conducted from November 2023 to January 2024. The samples were processed at the Department of Microbiology of the Faculty of Medicine of Málaga.

### Sample size

The sample size was calculated as per the following:

Total population (N): 1106 persons

Expected proportion (p): 0.03 (3% of the population is nasal carrier)

Confidence level: 95% (Z value = 1.96 for a 95% confidence interval)

Margin of error (E): 5% (or 0.05)


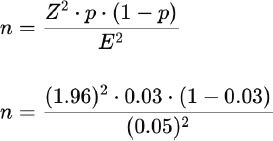


n = 44.64

The correction was applied to a population of 1106 individuals (applying finite population correction).


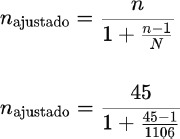


n = 43.3

The adjusted sample size for a population of 1106 individuals, with an expected proportion of 3% of different types of staphylococcus nasal carriers, a 5% margin of error, and a 95% confidence level, is approximately 43 individuals. The finite population correction has a minimal impact on the sample size compared to the initial estimate, given the relatively large population size.

A total of 53 veterinarians volunteered to participate in the study. Three of them were excluded from the study due to medical reasons that precluded them from meeting the inclusion criteria. The sample size was deemed sufficient for the initial phase of the study, which was conducted as a pilot.

## Inclusion criteria

The inclusion criteria for participation in the study were as follows: (1) Professional association membership, (2) active work, (3) absence of infectious pathologies or clinical health at the time of sample collection, and (4) signature of an informed consent form. Participants who received antibiotic treatment or were immunosuppressed upon sampling were excluded.

Veterinarians who consented to participate and met the inclusion criteria were summoned to the laboratory of the Department of Microbiology of the Faculty of Medicine of Málaga during January 2024. When samples could not be collected at the Faculty of Medicine, the technicians proceeded to the veterinarians’ work clinics. Samples were collected by two trained technicians following detailed instructions.

### Sample collection

The sample collection procedure included the following steps:

- Nasal sample: A swab was inserted into each nostril until it reached a depth of approximately 2–3 cm, rotated 360° several times in both nostrils and then removed.
- Hand swab: The skin is vigorously rubbed by the swab.

Following collection and identification of the samples (using a numerical system), they were stored in Stuart transport medium (Oxoid, Ltd., Hampshire, UK) for storage in the microbiology laboratory or transported, if necessary, until the time of analysis. All swabs were received at the laboratory within a maximum of 24 h.

### Sample processing: culture and identification

Upon arrival at the laboratory, nasal and hand swabs were seeded on mannitol agar with NaCl (MSA-Oxoid Hampshire, UK) to isolate, enumerate, and differentiate *Staphylococcus* spp. Following incubation at 37°C for 18–24 h, recovered colonies were evaluated for morphology and fermentative characteristics (colonial morphology, mannitol fermentation) and subjected to standard rapid screening techniques, including Gram staining, catalase, oxidase, and coagulase testing (Oxoid, Ltd., Hampshire, UK). The isolates were evaluated using API Staph (Biomerieux, Spain) and the Hampshire, UK, methods.

### Antimicrobial susceptibility testing

The isolated bacteria were tested for susceptibility to 11 antibiotics belonging to 5 classes. These antibiotics were selected based on their prevalence in veterinary practice, availability in the veterinary environment, and documented efficacy for treating bacterial infections in animals. The antibiograms were prepared using the disk-plate method on Mueller–Hinton agar (Oxoid, Ltd., Hampshire, UK). The antimicrobials tested were amoxicillin-clavulanic acid (AMC, 30 μg), amoxicillin (AML, 25 μg), ampicillin (AMP, 10 μg), oxacillin (OX, 1 μg), cephalexin (30 μg), cefoxitin (FOX, 30 μg), ciprofloxacin (CIP, 5 μg), enrofloxacin (ENR, 5 μg), clindamycin (DA, 2 μg), gentamicin (10 μg), and trimethoprim-sulfamethoxazole (SXT, 25 μg), all supplied by Oxoid, Ltd. The classification of isolates as susceptible, intermediate susceptible, or resistant was based on the European Committee on Antimicrobial Susceptibility Testing guidelines. *S. aureus* ATCC 12600, *S. epidermidis* ATCC 14990, *S. haemolyticus* ATCC 29970, and *S. pseudintermedius* ATCC 49051 were used for quality control.

### Statistical analysis

All data were recorded using Microsoft^®^ Excel 2019 (Microsoft Corporation, Redmond, Washington, USA). Descriptive statistics were used to analyze the collected data. The Mann–Whitney U test, Kruskal–Wallis test, Chi-square, and Fisher exact tests were used to analyze individual microbiological results, depending on the type of data. In addition, only significant factors from the univariate analysis were subjected to multiple comparisons with Bonferroni correction. All statistical analyses were performed using the R Programming language version 4.2.0 (https://www.r-project.org) with a 95% confidence interval, and p < 0.05 was considered significant.

## Results

A total of 50 veterinarians, representing 4.5% of the 1106 registered professionals in the province of Malaga, met the eligibility criteria and volunteered to participate in the study. The participants, comprising 36 men and 14 women, were employed at 31 veterinary clinics in Malaga. Among these healthy volunteers, various age groups ranging from 24 to 67 years (median 34.5; mean age 36.9) were represented. Regarding the type of practice, 76% (38) of veterinarians indicated that they are working in small animal practice, whereas 24% (12) are working in mixed practice.

The isolates recovered from the hands and nostrils are presented in Table-1. The concomitant *S. aureus* and *S. epidermidis* isolates are provided in Table-2. *S. pseudintermedius* strains were not isolated from the nostril and hand samples of the study participants. In two individuals, the analysis of both hand and nostril samples indicated non-culturable microorganisms.

**Table-1:** Presence of *Staphylococcus* species on hands and nasal passages.

Location	*Staphylococcus* spp.	Positive samples	Percentage
Hands	*S. aureus*	15	30
	*S. epidermidis*	15	30
	Other *Staphylococcus*	16	32
Nasal passages	*S. aureus*	16	32
	*S. epidermidis*	32	64
	*S. aureus* and *S. epidermidis*	4	8
	Other *Staphylococcus*	6	12

*S. aureus*=*Staphylococcus* aureus, *S. epidermidis*=*Staphylococcus epidermidis*

**Table-2:** Concomitant Isolation of *S. aureus* and *S. epidermidis*.

Nasal carriers (spp.)	Observation	Number of cases	Percentage
*S. aureus*	*S. aureus* isolated from hands	6	37.5
(16 carriers)	*S. epidermidis* isolated from hands	4	25
*S. epidermidis*	Colonized with *S. aureus* on the hands	11	34.4
(32 carriers)	Colonizing with other *Staphylococci* on the hands	10	31.3
	*S. epidermidis* was also isolated from the hands	9	28.1

*S. aureus*=*Staphylococcus* aureus, *S. epidermidis*=*Staphylococcus epidermidis*

The statistical analysis revealed no significant differences between males and females in the prevalence of nasal carriage of *S. aureus*. However, a higher frequency of nasal carriage of *S. epidermidis* was observed in females than in males among veterinarians (p = 0.044).

**Figure-1:**
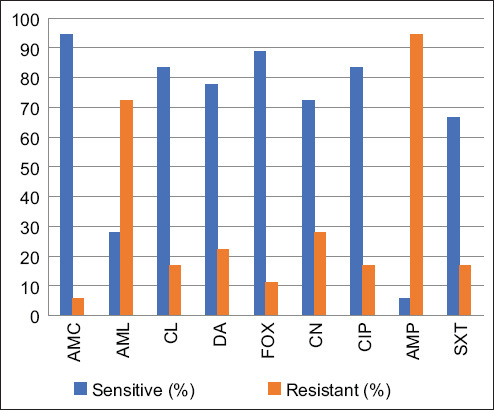
Susceptibility of *Staphylococcus aureus* to the antibiotics tested. AMC=Amoxicillin-clavulanic acid, AML=Amoxicillin, CL=Cephalexin, DA=Clindamycin, FOX=Cefoxitin, CN=Gentamicin, CIP=Ciprofloxacin, AMP=Ampicillin, SXT=Sulfamethoxazole trimethoprim.

Figure-1 shows the susceptibility and resistance rates of *S. aureus* to antibiotics. A high proportion of *S. aureus* strains isolated in the study group was AMP resistant (94.4%) and AML (72.2%). Regarding MRSA, two strains (11.1%) were identified among the 18 analyzed. In both cases, *S. aureus* was simultaneously isolated from the nostrils and hands.

## Discussion

Veterinary clinical practice plays a central role in linking human and pet health, with the potential for professionals to carry multi-resistant bacteria that pose risks to humans and animals [32].

Nasal and hand contamination by multi-resistant bacteria among veterinarians represents a public and animal health problem. Like other health professionals, veterinary professionals are at risk of colonization by these bacteria because of their continuous exposure to animals and potentially contaminated environments [33]. These conditions not only put the health of professionals at risk but also facilitate the transmission of infections to animals and humans, especially in the context of close contact and the management of zoonotic infections.

Although some studies have evaluated the potential risks [4, 34–36], there is a lack of research examining these risks among veterinary professionals. Data availability is critical for implementing control measures and hygiene practices that mitigate these risks.

This study evaluated the results of colonization by different Staphylococci for the first time in a sample of clinical practice veterinarians from the province of Málaga. Our results do not show differences with colonization in the general population, and they are similar to those reported by previous studies [4, 34–36].

None of the samples showed growth of *S. pseudintermedius*. The antibiotic resistance patterns observed in this study were also highly consistent with those documented in the literature in populations without specific exposure to companion animals.

A recent study conducted in Italy [37] revealed that 55.9% (19/34 samples) of veterinarians were found to be carriers of *Staphylococcus spp*., whereas 44.1% (15/34 samples) showed no growth of *Staphylococcus*. Of the positive cases, *S. aureus* was isolated in 52.6% (10/19 strains), whereas 26.3% (5/19 strains) exhibited growth of *S. pseudintermedius*. In all isolates, high resistance values were observed against β-lactams, with resistance rates of 89.4% for AMP and penicillin, followed by AMC (78.9%), OX (57.8%), and FOX (52.6%). Among the non-β-lactam antibiotics, resistance to erythromycin (73.6%) and DA (57.8%) were observed. Furthermore, additional resistance was observed to antibiotics belonging to the following classes: (i) Fluoroquinolones exhibited 47.3% and 36.8% resistance to ENR and CIP, respectively; (ii) tetracyclines demonstrated the same level of resistance to both doxycycline and tetracycline (42.1%); and (iii) sulfonamides exhibited 36.8% resistance to SXT [37].

This study revealed that 11.1% (2/18) of *S. aureus*-colonized individuals were MRSA. The results of the other studies are comparable. For example, a study by Neradova *et al*. [38] reported that the prevalence of nasal carriage of MRSA among veterinary professionals in the Czech Republic was 6.72%. Similar findings were reported by Hanselman *et al*. [39], who identified MRSA isolates in the nares of 6.5% of attendees at an international veterinary conference. The prevalence rate was 7.0% among veterinarians and 12.0% among technicians.

Finally, the findings of this study align with those previously documented for the general population regarding *S. aureus* and S. *epidermidis*. *Staphylococcus aureus* is commonly found on the skin and in the nose of about 30% of individuals [40], and permanent nasal colonization occurs in approximately 20% (range 12–30%) of individuals, with approximately 30% being intermittent carriers (range 16–70%) [41]. *S. epidermidis* is the most frequently isolated species from human epithelia [42].

It should be noted that this study is subject to certain limitations, namely the small number of participating veterinarians and the voluntary nature of their participation. It is also important to consider the absence of polymerase chain reaction-based techniques in this study as a further limitation. Our study was designed as a preliminary investigation to obtain an initial insight into the magnitude of clinically relevant *Staphylococcus* presence among healthy veterinarians in clinical practice. Furthermore, the *Staphylococcus* species included in the study were readily identifiable on the selective culture media used, which, combined with the biochemical tests performed and the use of API Staph test strips, provided high reliability in their identification.

## Conclusion

The analysis of samples from the volunteer veterinarians (obtained from the nostrils and hands) revealed the presence of *S. aureus* and *S. epidermidis* at proportions comparable to those observed in the general population. No strains of *S. pseudintermediu*s were found in either the nasal samples or hands of the study participants.

The colonization profiles of veterinary professionals were similar to those observed in the general population. Further research is required among veterinary professionals, companion animals, and their owners to gain a deeper understanding of the colonization processes and the pet-human interface within a “One Health” approach.

## Authors’ Contributions

FFG, RLG, EMM, and AJV: Conceptualized and designed the study and reviewed and edited the manuscript. FFG and EMM: Supervised field sampling, data collection, and laboratory work. RLG and AJV: Data entry, analysis, and interpretation, and participated in the preparation of the manuscript. All authors have read and approved the final version of the manuscript.

## References

[1] McEwen S.A, Collignon P.J (2018). Antimicrobial resistance:A one health perspective. Microbiol. Spectr.

[2] Palma E, Tilocca B, Roncada P (2020). Antimicrobial resistance in veterinary medicine:An overview. Int. J. Mol. Sci.

[3] Albrich W.C, Harbarth S (2008). Healthcare workers:Source, vector, or victim of MRSA?. Lancet Infect. Dis.

[4] Cuny C, Layer-Nicolaou F, Weber R, Köck R, Witte W (2022). Colonization of dogs and their owners with *Staphylococcus aureus* and *Staphylococcus pseudintermedius* in households, veterinary practices, and healthcare facilities. Microorganisms.

[5] Schmidt A, Bénard S, Cyr S (2015). Hospital cost of Staphylococcal infection after cardiothoracic or orthopedic operations in France:A retrospective database analysis. Surg. Infect. (Larchmt).

[6] Williams R.E.O (1963). Healthy carriage of *Staphylococcus aureus*:Its prevalence and importance. Bacteriol. Rev.

[7] Sivaraman K, Venkataraman N, Cole A.M (2009). *Staphylococcus aureus* nasal carriage and its contributing factors. Future Microbiol.

[8] Wertheim H.F.L, Melles D.C, Vos M.C, Van Leeuwen W, Van Belkum A, Verbrugh H.A, Nouwen J.L (2005). The role of nasal carriage in *Staphylococcus aureus* infections. Lancet Infect. Dis.

[9] Mulcahy M.E, McLoughlin R.M (2016). Host-bacterial crosstalk determines *Staphylococcus aureus* nasal colonization. Trends Microbiol.

[10] Brown A.F, Leech J.M, Rogers T.R, McLoughlin R.M (2014). *Staphylococcus aureus* colonization:Modulation of host immune response and impact on human vaccine design. Front. Immunol.

[11] Kluytmans J.A.J.W, Manders M.J, Van Bommel E, Verbrugh H (1996). Elimination of nasal carriage of *Staphylococcus aureus* in hemodialysis patients. Infect. Control Hosp. Epidemiol.

[12] Perl T.M, Cullen J.J, Wenzel R.P, Zimmerman M.B, Pfaller M.A, Sheppard D, Twombley J, French P.P, Herwaldt L.A (2002). Intranasal mupirocin to prevent postoperative *Staphylococcus aureus* infections. N. Engl. J. Med.

[13] Bode L.G.M, Kluytmans J.A.J.W, Wertheim H.F.L, Bogaers D, Vandenbroucke-Grauls C.M.J.E, Roosendaal R, Troelstra A, Box A.T.A, Voss A, Van der Tweel I, van Belkum A, Verbrugh H.A, Vos M.C (2010). Preventing surgical-site infections in nasal carriers of *Staphylococcus aureus*. N. Engl. J. Med.

[14] Nouwen J, Schouten J, Schneebergen P, Snijders S, Maaskant J, Koolen M, Van Belkum A, Verbrugh H.A (2006). *Staphylococcus aureus* carriage patterns and the risk of infections associated with continuous peritoneal dialysis. J. Clin. Microbiol.

[15] Garrouste-Orgeas M, Timsit J.F, Kallel H, Ben A.A, Dumay M.F, Paoli B, Misset B, Carlet J (2001). Colonization with methicillin-resistant *Staphylococcus aureus* in ICU patients:Morbidity, mortality, and glycopeptide use. Infect. Control Hosp. Epidemiol.

[16] Grundmann H, Aires-de-Sousa M, Boyce J, Tiemersma E (2006). Emergence and resurgence of methicillin-resistant *Staphylococcus aureus* as a public-health threat. Lancet.

[17] Stefani S, Goglio A (2010). Methicillin-resistant *Staphylococcus aureus*:Related infections and antibiotic resistance. Int. J. Infect. Dis.

[18] Tilahun B, Faust A.C, McCorstin P, Ortegon A (2015). Nasal colonization and lower respiratory tract infections with methicillin-resistant *Staphylococcus aureus*. Am. J. Crit. Care.

[19] Salgado C.D, Farr B.M, Calfee D.P (2003). Community-acquired methicillin-resistant *Staphylococcus aureus*:A meta-analysis of prevalence and risk factors. Clin. Infect. Dis.

[20] Mork R.L, Hogan P.G, Muenks C.E, Boyle M.G, Thompson R.M, Sullivan M.L, Morelli J.J, Seigel J, Orscheln R.C, Bubeck Wardenburg J, Gehlert S.J, Burnham C.A.D, Rzhetsky A, Fritz S.A (2020). Longitudinal, strain-specific *Staphylococcus aureus* introduction and transmission events in households of children with community-associated methicillin-resistant *S aureus* skin and soft tissue infection:A prospective cohort study. Lancet Infect. Dis.

[21] Ross Fitzgerald J (2009). The *Staphylococcus intermedius* group of bacterial pathogens:Species re-classification, pathogenesis and the emergence of methicillin resistance. Vet. Dermatol.

[22] Bannoehr J, Guardabassi L (2012). *Staphylococcus pseudintermedius* in the dog:Taxonomy, diagnostics, ecology, epidemiology and pathogenicity. Vet. Dermatol.

[23] Dos Santos T.P, Damborg P, Moodley A, Guardabassi L (2016). Systematic review on global epidemiology of methicillin-resistant *Staphylococcus pseudintermedius*:Inference of population structure from multilocus sequence typing data. Front. Microbiol.

[24] Talan D.A, Goldstein E, Staatz D, Overturf G.D (1989). *Staphylococcus intermedius*:Clinical presentation of a new human dog bite pathogen. Ann. Emerg. Med.

[25] Börjesson S, Gómez-Sanz E, Ekström K, Torres C, Grönlund U (2015). *Staphylococcus pseudintermedius* can be misdiagnosed as *Staphylococcus aureus* in humans with dog bite wounds. Eur. J. Clin. Microbiol. Infect. Dis.

[26] Somayaji R, Priyantha M.A.R, Rubin J.E, Church D (2016). Human infections due to *Staphylococcus pseudintermedius*, an emerging zoonosis of canine origin:Report of 24 cases. Diagn. Microbiol. Infect. Dis.

[27] Han J.I, Yang C.H, Park H.M (2016). Prevalence and risk factors of *Staphylococcus* spp. carriage among dogs and their owners:A cross-sectional study. Vet. J.

[28] Rodrigues A.C, Belas A, Marques C, Cruz L, Gama L.T, Pomba C (2018). Risk factors for nasal colonization by methicillin-resistant staphylococci in healthy humans in professional daily contact with companion animals in Portugal. Microb. Drug Resist.

[29] Moses I.B, Santos F.F, Gales A.C (2023). Human colonization and infection by *Staphylococcus pseudintermedius*:An emerging and underestimated zoonotic pathogen. Microorganisms.

[30] Severn M.M, Horswill A.R (2022). *Staphylococcus epidermidis* and its dual lifestyle in skin health and infection. Nat. Rev. Microbiol.

[31] Caneschi A, Bardhi A, Barbarossa A, Zaghini A (2023). The use of antibiotics and antimicrobial resistance in veterinary medicine, a complex phenomenon:A narrative review. Antibiotics.

[32] Gyles C (2009). Infection control in veterinary clinics. Can. Vet. J.

[33] Akwuobu C.A, Ngbede E.O, Mamfe L.M, Ezenduka E.V, Chah K.F (2021). Veterinary clinic surfaces as reservoirs of multi-drug-and biocide-resistant Gram-negative bacteria. Access Microbiol.

[34] Sakr A, Brégeon F, Mège J.L, Rolain J.M, Blin O (2018). *Staphylococcus aureus* nasal colonization:An update on mechanisms, epidemiology, risk factors, and subsequent infections. Front. Microbiol.

[35] Gómez-Sanz E, Torres C, Lozano C, Zarazaga M (2013). High diversity of *Staphylococcus aureus* and *Staphylococcus pseudintermedius* lineages and toxigenic traits in healthy pet-owning household members. Underestimating normal household contact. Comp. Immunol. Microbiol. Infect. Dis.

[36] Kottler S, Middleton J.R, Perry J, Weese J.S, Cohn L.A (2010). Prevalence of *Staphylococcus aureus* and methicillin-resistant *Staphylococcus aureus* carriage in three populations. J. Vet. Intern. Med.

[37] Nocera F.P, Pizzano F, Masullo A, Cortese L, De Martino L (2023). Antimicrobial resistance to *Staphylococcus* species colonization in dogs, their owners, and veterinary staff of the veterinary teaching hospital of Naples, Italy. Pathogens.

[38] Neradova K, Jakubu V, Pomorska K, Zemlickova H (2020). Methicillin-resistant *Staphylococcus aureus* in veterinary professionals in 2017 in the Czech Republic. BMC Vet. Res.

[39] Hanselman B.A, Kruth S.A, Rousseau J, Low D.E, Willey B.M, McGeer A, Weese J.S (2006). Methicillin-resistant *Staphylococcus aureus* colonization in veterinary personnel. Emerg. Infect. Dis.

[40] CDC Staphylococcus aureus Basics. Key Points.

[41] Piewngam P, Otto M (2024). *Staphylococcus aureus* colonisation and strategies for decolonisation. Lancet Microbe.

[42] Otto M (2009). Staphylococcus epidermidis - the 'accidental'pathogen. Nat. Rev. Microbiol.

